# Assessing the prevalence and risk factors of pre-diabetes among the community of Iganga municipality, Uganda: a cross sectional study

**DOI:** 10.1186/s13104-019-4589-1

**Published:** 2019-08-30

**Authors:** Christine Aramo, Anthony Peter Oyom, Emmanuel Okello, Victoria Acam, John Charles Okiria, Bashir Mwambi, Caesar Oyet

**Affiliations:** Clarke International University, P O Box 7782, Kampala, Uganda

**Keywords:** Pre-diabetes, Type 2 diabetes mellitus, Impaired glucose tolerance, Impaired fasting glucose, Iganga municipal

## Abstract

**Objective:**

The prevalence of pre-diabetes is increasing globally with more than 470 million people projected to develop pre-diabetes by 2030. In Africa, the average prevalence of pre-diabetes was estimated at 7.3% in 2015 and affected individual will develop type 2 diabetes mellitus within few decades. The aim of the study was to determine the prevalence of pre-diabetes and associated risk factors among residents of Iganga municipality. A cross-sectional study was conducted among males and females aged 13–60 years. District health office provided updated household list from which sampling of the villages was performed based on probability proportionate to population. Consented participants were prepared for the study, allowing fasting for 8 to 10 h before blood collection the next morning. Individuals with impaired fasting glucose, were subjected to OGTT.

**Results:**

130 participants were enrolled, of which 98 were women. The mean age of the participants was 35 years. The prevalence of pre-diabetes was 3.8%. The proportion of impaired glucose tolerance was higher in current smokers (p = 0.01), obese participants (p = 0.002) and hypertensive participants (p < 0.001). Prevalence of pre-diabetes is high in this community and is associated with current smoking, hypertension and high BMI.

## Introduction

Pre-diabetes is a state characterized by one or combinations of these factors; impaired fasting glucose (IFG), impaired glucose tolerance (IGT) and elevated glycated haemoglobin [1, 2]. It is seen as intermediate hyperglycaemia, which is not too high to be defined as diabetes mellitus but not low enough to be considered normal [3, 4]. This condition starts to develop early before the development of overt diabetes mellitus [5]. Numerically, pre-diabetes is defined as fasting blood glucose level of 6.1 mmol/L to 6.9 mmol/L with or glucose tolerance of 7.9 mmol/L to 11.0 mmol/L or glycated haemoglobin of 5.7% to 6.4% [6–11].

The prevalence of pre-diabetes is rising worldwide and it was estimated that by the year 2030, more than 470 million people will develop pre-diabetes [3]. In Africa, the prevalence of pre-diabetes could be even higher. From an estimated 26.9 million diagnosed with pre-diabetes in 2010 in Africa, there could be an estimated increase to 47.3 million pre-diabetes cases in Africa by the year 2030 [12]. In Uganda, a study conducted in rural Eastern Uganda in 2013 put the prevalence of pre-diabetes at 8.6% and that of diabetes mellitus at 7.4% using WHO criteria [13]. Because of the reduced health seeking behaviour of the rural community, the prevalence of pre-diabetes could be even higher in Uganda. Unfortunately, about 5% to 10% of those with pre-diabetes will progress to overt type 2 diabetes mellitus (T2DM) annually, therefore rendering pre-diabetes a risk factor for developing T2DM [3, 14, 15]. The progression to T2DM on this scale would strain Uganda’s health system which is already burdened with several infectious and non-communicable diseases. Besides, pre-diabetes is a risk factor for other metabolic disorders including insulin resistance, obesity, hyperlipidemia and heart related diseases [16–21].

Like with T2DM, there are a number of factors that seem to increase the chance of developing pre-diabetes. In a study among Swedish residents, high alcohol consumption was implicated as a risk factor for developing pre-diabetes [22, 23]. It seems that high alcohol consumption interferes with normal glucose regulation as it is known to alter the normal metabolism by depleting the body of NAD+ required for regulating fasting and non-fasting metabolism. Other risk factors for developing pre-diabetes are increased body mass index [23], family history of diabetes [24], advanced age [25] or less consumption of fruits and vegetables [23].

There is insufficient data on prevalence of pre-diabetes in Uganda; yet without prevalence data it is difficult to justify targeted screening for high risk persons. Additionally, the percentage of the population requiring long term care and the cost of detecting one high risk person is also unclear.

## Main text

### Materials and methods

This was a descriptive cross-sectional study conducted in Iganga municipality, located along the Kampala–Malaba highway about 150 km away from the capital city of Uganda. The study was approved by the Institutional Review Committee of Clarke International University.

#### Sample size calculation

According to previous study carried out in a similar location in 2013 [13] the prevalence of pre-diabetes was estimated at 8.6%. Using the formula for sample size calculation for proportion

$${\text{n}} = \frac{{{\text{Z}}^{2} {\text{P}}\left( {1 - {\text{P}}} \right)}}{{{\text{d}}^{2} }}$$

where: n = the desired sample size. Z = critical values of normal distribution at 95%, which corresponds to 1.96. P = the proportion of the target population estimated to have pre-diabetes. d = estimated margin of error 5%

$$\begin{aligned} {\text{n}} = \,& \frac{{ 1. 9 6^{ 2} 0. 8 6 { }\left( { 1- 0. 8 6} \right)}}{{\left( {0.0 5} \right)^{ 2} }} \\ {\text{n}} &= 121 \\ \end{aligned}$$

Adjusting for the sample collection errors and loss of samples, the sample size was adjusted by 5% to approximately 127 and rounded off to 130. The minimum number of participants required for the study was 130. A list of all the households was obtained from the district health and the local council V offices. Sampling of the villages was based on probability proportionate to population. 130 males and females aged 13 to 60 years were randomly sampled from a sampled household for the survey. Each day, after selecting the centre of the village, a bottle was tossed and a direction to the first household depended on the direction the bottle top faced. After sampling the first participant from the first household, the researchers took right direction from that house to the next household. The procedure was followed until the required sample size was met.

After obtaining a written informed consent, an appointment was given to the eligible participant and a clear instruction for fasting was provided. The following morning, measurements of height, weight and blood pressure (BP) were carried out. 4 mL of blood was collected from each participants in fluoride/oxalate bottles for fasting blood glucose and a structured questionnaires administered by a trained interviewer to collect information on risk factors for pre-diabetes. All the participants with fasting blood glucose above 6.1 mmol/L but below 6.9 mmol/L were given appointment for oral glucose tolerance test (OGTT). The procedure for the OGTT was as described previously [26]. Briefly, after an overnight fasting, blood sample was drawn from the participant and then 75 g of pure glucose was dissolved in 250 mL of pure drinking water, flavored with lemon juice. Participants took the preparation within 5 min after which blood samples were obtained at 30, 60, 90, and 120 min for the measurement of glucose. Body mass index (BMI) was then calculated as weight in kilograms divided by the squares of height in meters. Participants were then classified as underweight if BMI was < 18.5 kg/m^2^, normal weight 18.5–24.9 kg/m^2^ and overweight if BMI was 24.9–30 kg/m^2^ and obese if BMI was > 30 kg/m^2^. Two blood pressure (BP) measurements were taken 5 to 30 min apart with participant seated; using a calibrated BP TRANSTEK machine. The mean of the two measurements was then calculated. The BP of the participant was classified as hypertensive if the systolic BP > 140 mmHg and or diastolic BP > 90 mmHg. IFG and IGT were defined based on WHO criteria [11]. Data were double entered in Epi Data, cleaned and exported to STATA 10 (StataCorp, College Station, TX, USA) for analysis. The prevalence of pre-diabetes was calculated as the proportion of those participants classified as pre-diabetic against the total numbers of the participants. To determine the risk factors for pre-diabetes, we used prevalence rate ratios using modified poisson regression analysis model. 95% confidence level was used in the analysis and statistical significance was set at p < 0.05.

### Results

We recruited 130 participants aged 13 to 60 years, 98 (75.4%) of which were females and 32 (24.6%) were males. The median age was 34 years (IQR 17 years). Majority of the participants had less than primary school education [92 (70.8%)] and currently married [75 (57.7%)]. 74 (56%) were employed of which 53 (40.8%) were self-employed (Table 1).

**Table 1:** Demographic characteristics of the participants (n = 130)

Category	Frequency	Percentage
Education level		
Primary or less	92	70.8
Secondary education	25	19.2
More than secondary education	13	10.0
Marital status		
Never married	32	24.5
Married	75	57.7
Divorced	16	12.3
Widowed	7	5.5
Employment		
Employed	74	56.9
Not employed	7	5.4
Retired	49	37.7

The overall mean fasting glucose (FBG) level was 4.9 mmol/L (95% CI 4.9, 5.1 mmol/L). The mean FBG was 4.8 mmol/L (95% CI 4.8, 5.2 mmol/L) among males and 4.9 mmol/L (95%; CI 4.8, 5.2 mmol/L among females (p = 0.8). Majority of the participants 122 (93.8%) had normal blood glucose levels, 5 (3.9%) had impaired fasting glucose and the rest 3 (2.3%) had diabetes mellitus. Using WHO criteria the prevalence of pre-diabetes (IGT) was 3.9% (Table 2).

**Table 2:** Risk factors for the development of pre-diabetes (n = 130)

Variable	Normoglycaemia	Pre-diabetes	Diabetes	p value
Current smoker	4	1	1	0.01
Non smoker	126	4	0	
Alcohol intake	33	0	0	0.21
Non-alcohol intake	97	0	0	
Fruit intake	116	5	3	0.36
No fruit intake	14			
Moderate exercise	37	2	0	0.96
Intense exercise	93	0	0	
Fat consumption	107	5	3	0.02
No fat consumption	43	0	0	
Less than 2 days a week of vegetable intake	87	5	2	0.01
No veg. intake	43	0	0	
Age (> 40 years)	36	2	3	0.01
Age (< 40 years)	94	3	0	
Sex (females)	90	4	2	0.91
Sex (males)	40	1	1	

The proportion of impaired glucose tolerance was higher in current smokers (16.7%) compared to non-smokers (3.3%) (p = 0.01).

The mean BMI of the participants was 24 kg/m^2^ with a standard deviation of 4.7 kg/m^2^. 13 of our participants were obese of which 1 was male and 12 were females. Multivariate logistic regression shows obesity is associated with impaired glucose tolerance (p = 0.002).

The proportion of hypertension was 7.8%, of these, 1 participant had only systolic hypertension and 9 had diastolic hypertension with no participant with combined hypertension. A logistic regression examining the relationship between hypertension and impaired glucose tolerance shows a positive association between hypertension and impaired glucose tolerance (p < 0.001).

### Discussion

Diabetes mellitus incidence has increased lately with change in the usual trends; many people developing diabetes not only in high income countries, but also in middle and low income countries [27, 28]. The contributing factor to this change is the adoption of sedentary lifestyles and dietary change to more fatty and greasy foods. The main focus of clinical practice is on management of diabetes; overlooking the fact that before overt diabetes, affected individuals would have had pre-diabetes for many years. Most of the pre-diabetic patients develop overt diabetes in 5 to 10 years and are at risk of complications including premature cardiovascular diseases [29]. Few studies have attempted to estimate the prevalence of pre-diabetes in sub-Saharan Africa. Our study estimated that 3.9% of the residents of Iganga municipal aged 13 to 60 years have pre-diabetes. This result is much lower than that of an earlier study carried out in a similar setting in the rural parts of Mayuge and Iganga where the prevalence of pre-diabetes was 8.6% [13]. This difference is expected since in the current study, the age of the participants spanned from 13 to 60 years, with inclusion of individuals of low risk and high risk age groups. In the Mayuge-Iganga study, participants were recruited only from the high risk age group of 35 to 60 years. Being peri-urban, our participants can be seen as more knowledgeable of factors that would modify their lifestyle in an attempt to reduce the incidence of diabetes mellitus and pre-diabetes.

Several risk factors exist which link individuals to the development of pre-diabetes and diabetes mellitus [27, 30]. According to our findings, the factors which were positively associated with pre-diabetes mellitus were current smoking, hypertension and high BMI. These are modifiable risk factors that can be averted. Persons with these risk factors should therefore be targeted for intense life style education that emphasizes increased physical activity, health education on dangers of smoking, self-monitoring of body weight and periodic monitoring of blood glucose level which can be integrated into outpatient department services.

The prevalence of undiagnosed diabetes mellitus among the participants was 2.3% suggesting that there are a number of individuals who do not know their glycaemic status, or have undiagnosed diabetes mellitus. These are individuals who if not detected early could develop diabetes mellitus, suffer reduced quality of life or even die as a result of complications associated with diabetes mellitus.

### Conclusion

The prevalence of pre-diabetes is high among the residents of Iganga municipality, and the incidence of pre-diabetes is associated with current smoking, hypertension and BMI > 25 kg/m^2^.

## Limitations of the study

The study enrolled few participants and association of pre-diabetes with proposed risk factors would have been difficult to establish. Participants’ preparation especially on the non-observed fasting might have affected the overall prevalence of pre-diabetes and conclusion was based on only what was collected from the field visit. Since most of the men were at work, few who were at home were enrolled for the study. This over represented females who were most of the time at home.

The study enrolled few participants and this would have affected the proportions of IGT, diabetes and pre-diabetes. However, the results show a trend which can guide further studies to establish not only prevalence but also related associations.

## Data Availability

The datasets used and/or analysed during the current study is available and will be provided by the corresponding author on reasonable request.

## Abbreviations

IQR: interquartile range
BMI: body mass index
OGTT: oral glucose tolerance test
T2DM: type 2 diabetes mellitus
IFG: impaired fasting glucose
IGT: impaired glucose tolerance
BP: blood pressure
